# kpath: integration of metabolic pathway linked data

**DOI:** 10.1093/database/bav053

**Published:** 2015-06-08

**Authors:** Ismael Navas-Delgado, María Jesús García-Godoy, Esteban López-Camacho, Maciej Rybinski, Armando Reyes-Palomares, Miguel Ángel Medina, José F. Aldana-Montes

**Affiliations:** ^1^ Departamento de Lenguajes y Ciencias de la Computación, Universidad de Málaga, Andalucía Tech, Ada Byron Research Building, E-29071 Málaga, Spain, ^2^ Departamento de Biología Molecular y Bioquímica, Facultad de Ciencias, Universidad de Málaga, Andalucía Tech, and IBIMA (Biomedical Research Institute of Málaga), E-29071 Málaga, Spain and ^3^CIBER de Enfermedades Raras (CIBERER) E-29071 Málaga, Spain

## Abstract

In the last few years, the Life Sciences domain has experienced a rapid growth in the amount of available biological databases. The heterogeneity of these databases makes data integration a challenging issue. Some integration challenges are locating resources, relationships, data formats, synonyms or ambiguity. The Linked Data approach partially solves the heterogeneity problems by introducing a uniform data representation model. Linked Data refers to a set of best practices for publishing and connecting structured data on the Web. This article introduces *kpath*, a database that integrates information related to metabolic pathways. *kpath* also provides a navigational interface that enables not only the browsing, but also the deep use of the integrated data to build metabolic networks based on existing disperse knowledge. This user interface has been used to showcase relationships that can be inferred from the information available in several public databases.

**Database URL**: The public Linked Data repository can be queried at http://sparql.kpath.khaos.uma.es using the graph URI “www.khaos.uma.es/metabolic-pathways-app”. The GUI providing navigational access to *kpath* database is available at http://browser.kpath.khaos.uma.es.

## Introduction

Over the last two decades, the biological database community has witnessed a rapid growth in the number of available data sources. The growth is a result of an enormous increase in the availability and accessibility of biological data. In the beginning, the information was used by a few specialized disciplines but today, these databases have become essential resources used by scientists around the world.

In the metabolism field, there are many databases, such as Kegg (1), whose principal components are pathways (of the most known biochemical and regulatory networks) displayed in graphical diagrams. Brenda (2) is one of the most prominent available information systems, which provides biochemical and molecular information about classified enzymatic activity. Reactome (3) captures all the known chemical reactions and pathways of different organisms. Sabio-rk stores information about biochemical reactions and their kinetic properties. It can be leveraged to support biochemical networks analysis in terms of comparing reactions, their kinetic conditions and information related to cellular locations, tissues and organisms (4). The Human Metabolome Database (HMDB) is one of the most complete databases with curated information about human metabolism (5). This database contains a collection of metabolome data from mass spectra (MS) and nuclear magnetic resonance (NMR) analyses using human samples. BioCyc is a database which attempts to collect genome/pathway databases (PGDBs) from eukaryotic and prokaryotic species whose sequencing process is complete (6). This collection includes 5500 genome/pathway databases that are in the process of literature-based curation (5493 out of 5500) and those that have been involved in the curation process for at least a year (7 out of 5500) such as Metacyc (6), HumanCyc (7), PlantCyc (8), AraCyc (9), LeishCyc (6), TrypanoCyc (10) and YeastCyc (11). MetaCyc is a highly curated resource, which includes metabolic pathways of organisms that are presented in the literature. HumanCyc provides a genome-based view of human pathways and essential dietary nutritional requirements, placing genes in a metabolic pathways context. PlantCyc compiles information about plant metabolic pathways from databases such as MetaCyc, AraCyc, RiceCyc (12) and MedicCyc (13). This database stores metabolic pathways which are involved in primary and secondary metabolism providing information about rare and valuable compounds. The AraCyc is another plant-related database which stores pathway information from literature, about *Arabidopsis* displaying the data on a user-friendly interface. This database is comparable in terms of annotation quality with EcoCyc, a database that has been focused on collecting *Escherichia* *coli* information since mid-1990 (14). LeishCyc is a genome/pathway database for protozoan parasite *Leishmania* *major* with information on gene, gene products, pathways and reactions presented in the existing literature. TrypanoCyc is another database similar to LeishCyc, which describes the metabolic network of *Trypanosoma brucei,* a protozoan responsible for human and animal disease African trypanosomiasis. Finally, the Yeast Pathways database is a manually curated collection of metabolic and enzyme pathways of the *Saccharomyces cerevisiae* organism.

Many scientists use these databases on a daily basis in order to extract the biological information necessary for their work. As of now, some of the databases are partially interlinked (e.g. Kegg records have links to Brenda, Uniprot, etc.). This level of interoperability is sufficient for a simple navigational search, but does not allow a federated search across different resources. As a result, users need to perform many independent searches when they seek cross-database information. For example, if a user wants to see all the pathways from Kegg and Reactome, which involve a specific metabolite, they would have to run two independent searches for each of the databases. Even then, the actual overlap of those two sets of results would not be very clear. Furthermore, Stobbe *et al*. (15) recently carried out an assessment based on the available human pathway databases. The results of this study show that the data overlap of networks reconstructed with different databases, especially at the reaction level, was surprisingly low and therefore, the level of database agreement is deemed to be low as well. This study stresses the importance of the integration based on the use of standard data publishing practices. It also shows how important it is to enable an integrated search tools to retrieve full relevant information.

To address the problem of information integration, the Semantic Web community led by the W3C community proposed a set of standards such as the Resource Description Framework (RDF) which is a data model based on subject-predicate-object logical statements called triples (16). Since 2007, there has been a lot of effort dedicated to providing data sets from different areas using Semantic Web technologies. In this context, a set of best practices has been proposed for sharing, publishing and connecting data, information and knowledge by using RDF and URIs. These practices are known as Linked Data principles (17).

The move towards the publication and linking of data has been continuously growing and the number of documents and resources stored in the Linked Data Cloud (18) has grown to 900 129 documents and 8 038 396 resources in 2014 (latest update in 2014). In the early days of Linked Data, the RDF information published in Life Sciences increased enormously due to the output of projects such as Bio2RDF (19) and Linked Life Data (20). Bio2RDF is a semantic web database designed to solve the integration problem in the Life Sciences area. This mash-up system offers a web interface in which users can explore RDF triples from different data sources such as Biomodels (21), DrugBank (22), and Gene Ontology annotations (23). Linked Life Data is yet another platform which stores billions of biomedical and Life Sciences RDF triples. It integrates information from Pubmed (http://www.ncbi.nlm.nih.gov/pubmed), UMLS (24), Entrez-Gene (25) and Open Biological and Biomedical Ontologies (OBO) (26).

However, the use of Linked Data to distribute and provide access to public biological data and several well established databases provide their own points of RDF information access (SPARQL endpoints) (19, 27, 28). They support the access to these data using the SPARQL query language.

In Life Sciences, one of the main integration problems is related to the complexity of biological data. This feature makes it difficult to develop ‘wide-span’ applications for end-users. This article presents an integration solution in the context of the metabolic pathways that use Linked Data as a source of information. As a result of this process, *kpath* (*Khaos Metabolic Pathways*) database has been published. On top of the database, we have implemented *kpath Browser* with functionalities such as navigation, search and visualization to make the integrated data easily accessible to users with a biological background.

The data generated from genomics using next-generation sequencing techniques and metabolomics has increased in the last few years. For this reason, there has been a need to develop new applications to visualize the information related to the metabolic pathways and genes involved in the metabolism. These applications can be divided into two groups: the first group is composed of those databases that provide visualization of indexed chemical reactions and the second group includes web-based or desktop software that visualizes metabolic pathways. For example, in the first group, Kegg and Ecocyc databases only display pathways whose information is stored in the database. These database tools which visualize pathways are limited in features such as pathway editing, query pathways, etc. The second group of applications provides some of the aforementioned features that are not present in the first group of tools.

In the second group of applications, those known as web-based or desktop applications, Cytoscape (29) is a tool that represents biological pathways and covers a wide set of features such as pathway editing, exporting of pathway representation to different formats, etc. In fact, plug-ins for the Cytoscape application have been developed. MetScape 3 (30), is one such plug-in and visualizes metabolic pathways through the integration of three databases such as HUMDB (31), EHMN (32) and Kegg. This allows users to query and retrieve useful information to be analysed. However, despite the fact that MetScape is included in the Cytoscape functionalities, this application only integrates information from three organisms (human, rat and mouse) thereby limiting the information that can be queried and retrieved by users. Due to the existing limitations of this second group, the *kpath* database links genetic and metabolic information from Kegg, Uniprot and Reactome (3, 33, 34) databases by using Linked Data technology and all metabolic data from the organisms included in Kegg.

The *kpath* database itself can be accessed through the SPARQL endpoint, which is suitable not only for users that run their own queries, but also for the programmatic access of custom made applications that can be built to take full advantage of the service.

We also provide a specialized client, *kpath Browser* (http://browser.kpath.khaos.uma.es), which is an example of such an application, to access and exploit these integrated data. The main goals of this tool are:

- Provision of an easy-to-use tool for accessing the integrated *kpath* database without prior knowledge of SPARQL.
- Provision of a set of features to study the different pathways at different levels of detail, including at least the most common functionalities of other client tools (see Table 1); novel functionalities will also be of interest, such the possibilities to analyse the relationships between different pathways.
- Provision of the fine grain information (at the level of pathway components) inside the same client tool without needing to browse other client tools or databases; i.e. instead of providing links to external sources, the information is integrated and shown in the same tool.

The *Kpath Browser* covers most of the commonest functionalities and provides an advanced use of the data able to take advantage of the integration process. A comparative study has been performed with related tools based on the analysis of several dimensions, see Table 1. The detailed description of the dimensions of Table 1 has been included in supplementary material (see online supplementary file S1).

**Table 1.** Comparison of *kpath* Browser features with those of related tools

	Reference information	Pathway Editor Tool	Multiple species	Facilitate sharing across group members	Attached source information on nodes and edges	Manipulate node properties	Pathway comparison/alignment	Multiple-linked views	Zooming	Query Pathways	Genetic information and pathways	Build history of edited pathways	Update of Database	Building network from node list(s) by search	Integration of updated data from multiple sources	Export to standard formats
Kegg^a^	✓		✓										✓			
Biocarta	✓		✓										✓			
EcoCyc	✓												✓			
Pathway Editor^b^		✓	✓		✓										***N/A***	
PathwayAssist			***N/A***		✓	✓				✓				✓	***N/A***	✓
GenePath			***N/A***			✓								✓	***N/A***	✓
GeneMAPP			***N/A***	✓	✓	✓							✓		***N/A***	✓
Cytoscape	***N/A***	✓	***N/A***	✓	✓	✓	✓		✓	***N/A***	✓		***N/A***		***N/A***	✓
Knowledge Editor															***N/A***	✓
Biological Story Editor				✓								✓			***N/A***	
Patika			***N/A***				✓	✓		✓			✓		***N/A***	✓
Genies			✓												✓	
Vector PathBlazer	✓					✓							✓	✓		✓
MapMan											✓					✓
Pubgene	✓		✓	✓				✓							✓	
MetScape 3	✓	✓			✓	✓	✓		✓		✓			✓	✓	✓
MetDraw			***N/A***	✓		✓										
GeneSpring								✓			✓				***N/A***	
GenePath																✓
Chibe	✓		✓	✓		✓	✓			✓						✓
PCViz	✓			✓	✓				✓	✓	✓		✓	✓		✓
STKE	✓		✓				✓									
KeggScape	✓	✓	✓	✓	✓	✓	✓		✓	✓	✓		✓	✓		✓
kpath Browser	✓	✓	✓	✓	✓	✓	✓	✓	✓	✓	✓	✓	✓	✓	✓	✓

^a^Cells of this group correspond to database websites where pathways can be visualized.

bCells of this group correspond to visualization tools.

The article is divided as follows: System and methods section describes the process to provide access to heterogeneous biological information and the results obtained. The kpath user interface section presents a detailed description of the navigational interface of *kpath* database to access this integrated data, how it can be used and how the usage scenario behaves in comparison with manual access to the same. Use cases section describes several use cases with relevant biomedical implications. Conclusion section presents the conclusions resulting from this integration work and the consequent improvements to be implemented in the future.

## System and methods

**Figure 1.**
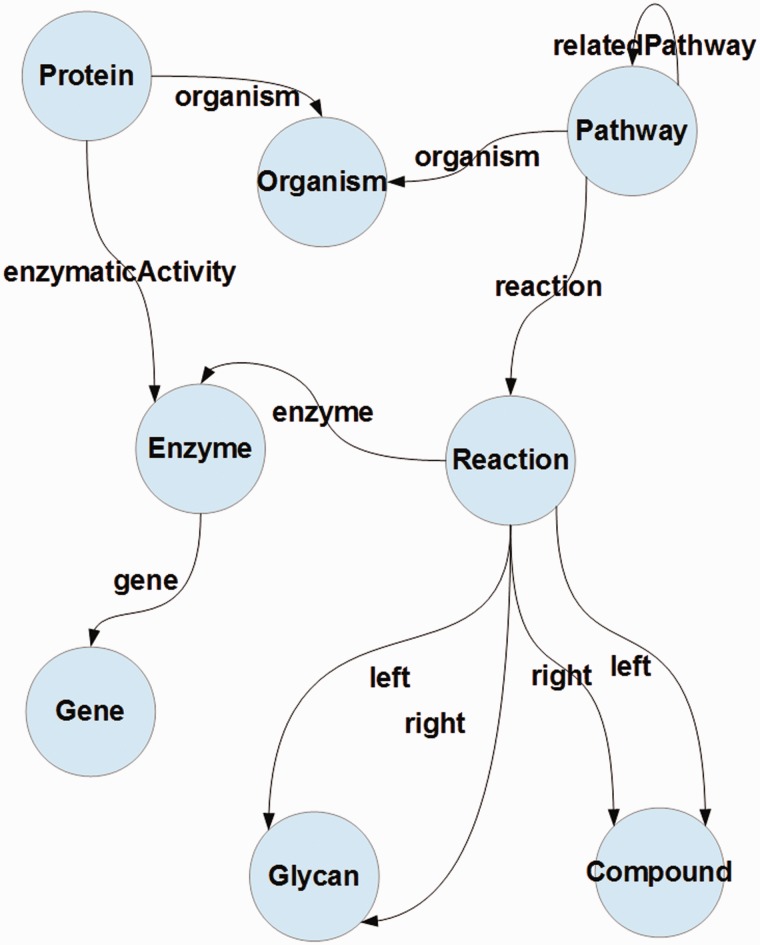
A conceptual representation of the data model proposed for the RDF pathway database. Circles denote concepts and links denote properties; concepts on the respective ends of a link (property) denote its domain and range.

In this article, we present an approach to integrate pathway data from four different Linked Data repositories. *kpath* takes the Bio2Rdf Kegg's data as the core, which is then extended with organism data from NCBI Taxonomy (35) and Protein data from SwissProt (36), as well as with related pathway data extracted from Bio2RDF Reactome distribution (3). The integration process is based on a simple ontology whose high level view is shown in Figure 1.

Table 2 shows the data properties of each of these concepts and their provenance. Pathway information has been manually extended with information on the metabolism when known.

**Table 2.** Data properties and their provenance (prefix khaos-pathways omitted for readability)

Concept	Property	Provenance
Compound	Name	Kegg and Reactome	
	synonym	Kegg	
	Formula	Kegg	
	Mass	Kegg	
Enzyme	Name	Kegg	
	Synonym	Kegg	
	Ecnumber	Kegg	
Gene	Id	Kegg	
Glycan	Name	Kegg	
	Synonym	Kegg	
	Formula	Kegg	
	Mass	Kegg	
Pathway	Name	Kegg and Reactome	
	Metabolism	Own Data	
Protein	Name	Uniprot/Swissprot
	Synonym	Uniprot/Swissprot
	UniprotID	Uniprot/Swissprot
	GeneName	Uniprot/Swissprot
	Keyword	Uniprot/Swissprot
	Comment	Uniprot/Swissprot
Organism	Name	NCBI taxonomy
	Synonym	NCBI taxonomy
	KeggCode	Kegg
	Comment	NCBI taxonomy
Reaction	Name	Kegg and reactome

The *kpath* data structure resembles one of the Bio2Rdf Kegg distributions, but with Organism concept instances compiled from the Taxonomy database, as the organism information was missing from the Bio2Rdf distribution. Additionally, we have added a Protein concept with different semantics to the Protein concept of Bio2Rdf data. In this case, the Protein concept refers to the one used in the SwissProt data.

Table 3 shows a summary of the concepts, total number of instances, average of edges and literals (that are not resources such as strings or numbers) per instance.

**Table 3.** A summary of nodes and edges stored in *kpath* RDF graph

Concept	No. of entities	Outgoing links per entity	Literals per entity
pathways:Compound	24 708	–	3.9
pathways:Enzyme	4245	105.34	6.81
pathways:Gene	689 997	–	2
pathways:Glycan	10965	–	2.1
pathways:Organism	2278	–	3.69
pathways:Pathway	83 097	16.36	2.01
pathways:Protein	538 849	1.22	16.95
pathways:Reaction	12 815	6.68	1.87

The Concept column represents all types (classes) stored in the RDF graph of the endpoint. The no. of entities represents the number of resources included in each type (e.g. in the pathway class). The third column represents the average of edges (links) in the graph. The fourth column represents the average of literals per resource.

Given the structure presented in Figure 1, it can be observed that there are five important merge points for these data, i.e. relationships between proteins and organisms, pathways and organisms, proteins and enzymes. Furthermore, compounds and enzymes can be used to relate pathways from different data sources, which in turn leads to matching reactions (through matching their components).

Links between pathways and organisms were established through the Kegg organism codes included in pathway identifiers and their relation to NCBI Taxonomy identifiers.

For example the ‘hsa’ code from Kegg corresponds to NCBI's 9606 (*Homo sapiens*).

The relationships between proteins extracted from SwissProt and organisms in the NCBI Taxonomy were extracted from the SwissProt data. For example, SwissProt data on GCSP_HUMAN contains an explicit reference to the 9606 taxon.

Links between proteins and enzymes were related with the SwissProt data through the E.C. Numbers. Thus, the information given in this database as a literal was used to determine the relationships between proteins and enzymes. For example GCSP_HUMAN synonym ‘1.4.4.2’ would lead to establishing a direct link between <khaos-pathways:GCSP_HUMAN> and <http://bio2rdf.org/ec:1.4.4.2>. The structural distinction between proteins and enzymes originates from a semantic mismatch between SwissProt and Kegg. The biggest difference between these representations is that Kegg instances represent an abstract protein concept with the same enzymatic activity across different organisms, whereas SwissProt instances represent proteins and their functions within specific organisms. For example <khaos-pathways:GCSP_HUMAN> and <http://bio2rdf.org/ec:1.4.4.2>.

**Figure 2.**
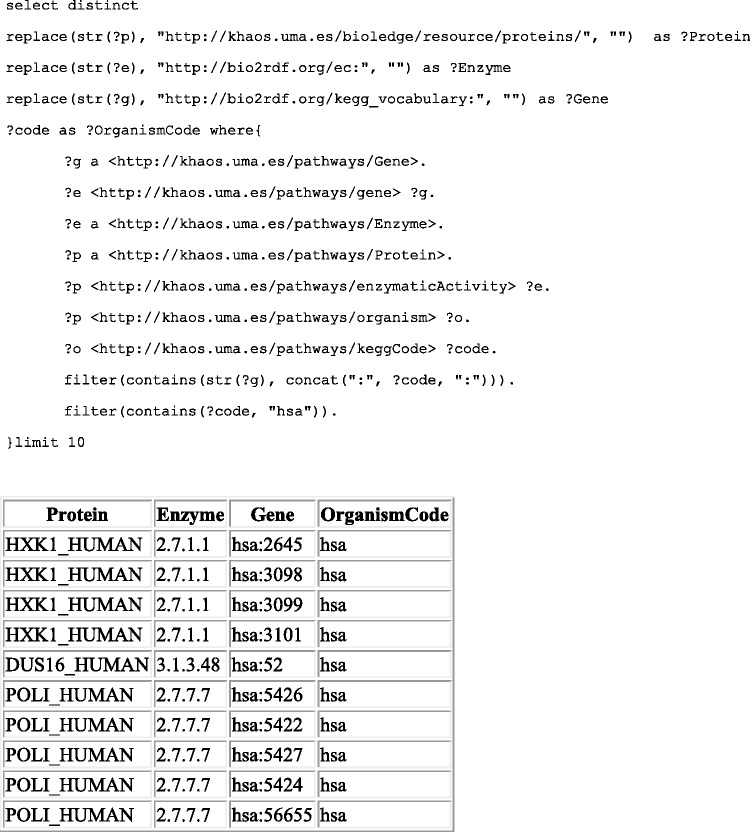
The top of the figure shows the SPARQL query used to get Kegg genes and SwissProt proteins linked by their corresponding organism codes and EC numbers. Results are shown at the bottom of the figure.

It is worth noting, that the data structure used by *kpath* also provides an indirect mapping (via linked enzymes) between SwissProt proteins end Kegg genes. Therefore, it is possible to query Kegg gene codes from certain Uniprot proteins. An example query with its results is presented in Figure 2.

Metabolic pathways from Reactome were also included in a way corresponding to the simple data structure presented above. In order to include new pathway data, the imported pathways have been represented as a set of biochemical reactions, which, due to the highly general nature of our model, implies a simplification for the majority of relevant datasets.

The instance matching approach we have proposed is based on relating basic concepts, i.e. compounds and enzymes, thus enabling querying for Kegg and Reactome pathways that share common components. Enzymes and enzyme groups can be easily connected through the E.C. numbers, while equivalence relationships for compounds were found through common CHEBI references found in the original datasets (Kegg and Reactome). As a result more than 25 K Reactome instances were mapped to a set of nearly 1 K Kegg compounds (many-to-one mappings due to different semantic scope of the original concepts). Matching those basic pathway components leads to matching specific reactions with over 3.5 K (out of ca. 8 K) reactions from Reactome mapped to their Kegg counterparts. In the case of Reactome instances that can be mapped to Kegg instances, we have merged the Reactome data into Kegg data, while maintaining a separate namespace. For example, if we discover that object R from Reactome has an equivalent K in Kegg, we simply substitute the identifier of R with the identifier of K wherever it appears within the Reactome extracted data. At the same time we keep track of data provenance by putting the Reactome data in a separate namespace, so that we can either include or exclude the Reactome data from our queries. The effect of this implementation choice can be seen in the pathway browser. It is worth noting, that only metabolic pathways were incorporated from Reactome.

The integrated data is available through a public SPARQL (Virtuoso) endpoint at http://sparql.kpath.khaos.uma.es. Users can run SPARQL queries, defined or parameterized queries using the Bioqueries platform (37) and retrieve the results in different standard formats, such as different serializations of the RDF graphs (RDF/XML, N-Triples, Turtle) or JSON, using the local vocabulary or BioPAX Level 3 (38).

## The kpath user interface

**Figure 3.**
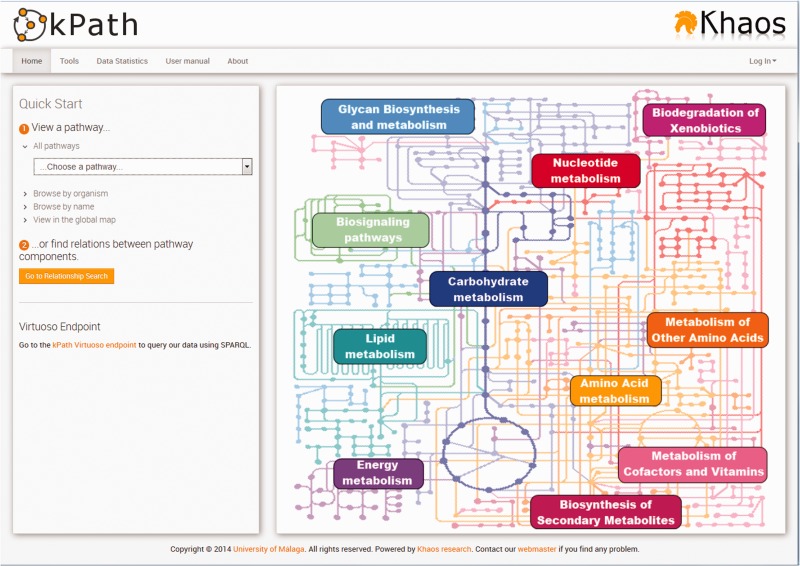
kpath Browser user interface to search for pathways.

The *kpath Browser* is publicly available through a Web interface, whose main page shows some options and links to quickly navigate to the different tools provided. As an entry point, the user can search a pathway using simple filters or the global metabolic map (Figure 3). The three main tools are: the Pathway Graphical Viewer, the Pathway Graphical Editor and the Relationship Search Tool. These three tools have a similar interface appearance. The Pathway Graphical Viewer and Pathway Graphical Editor are complementary to each other and have many characteristics in common. The Pathway Graphical Viewer allows visualizing a pathway as a graph, representing its components (reactions, metabolites, enzymes and genes) as different types of nodes. This interface also allows us to visualize more than one pathway at a time with two different view modes: one combining the common elements in more than one pathway in order to show them as a new bigger pathway, or to show them separately, marking the common nodes with union lines.

At any time, the Pathway Graphical Editor can be called using one (or all) of the pathways that are being displayed in the Pathway Graphical Viewer. The pathway components can be edited and saved as a new user pathway. The custom pathways can be stored so they can be loaded later. This new custom pathway, if saved, can also be used for comparisons in the Pathway Graphical Viewer.

The Relationship Search Tool allows some components to be selected from the integrated data or, using a file, search for some of them. After choosing a set of components (enzymes, metabolites or genes), the tool can be executed to find all the existing relations between each pair of the selected components.

## Use cases

This section shows how *kpath Browser* can be used to discover relevant knowledge from the integrated data through the description of two easy use cases regarding different pathological conditions.

One of the most interesting features of the *kpath Browser* is its capability to infer relationships from data. Many of these inferences correspond to new or not easily accessible information from biological databases by current standard methods. This situation demands improved biological data handling for users. For this reason, we have focused on these inferences to illustrate how data integration based on linked data technology provides comprehensive relationships and potential biomedical applications. To this end, we used *kpath Browser* to study biochemical reactions in two different medical conditions.

**Figure 4.**
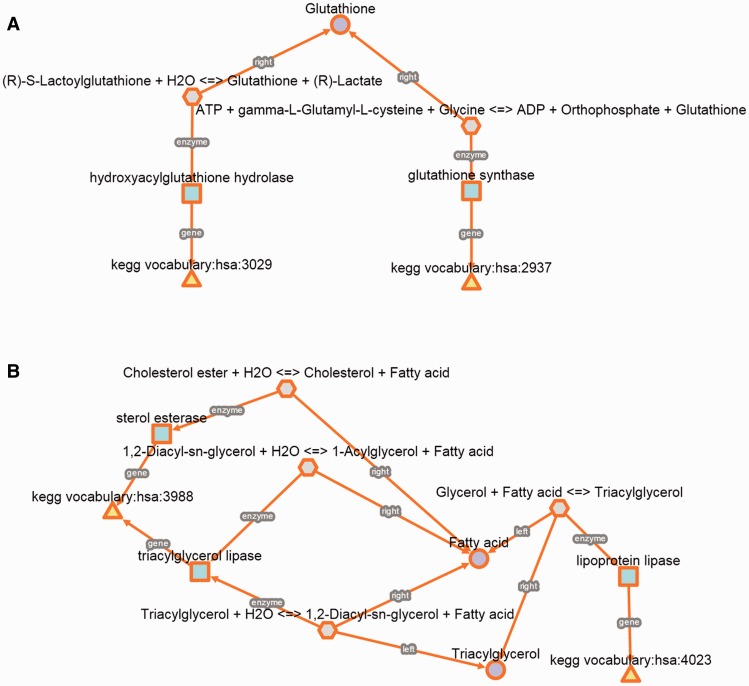
Detailed biochemical reactions and associated components for each case of use. (**A**) Glyoxylase deficiency. (**B**) Hypercholesterolemia.

In a first example, we studied the biochemical reactions between genes genetically related to a very specific phenotype such as glyoxalase deficiency. This phenotype is caused by mutations in glutathione synthetase (GSS) or hydroxyacylglutathione hydrolase (HAGH), but it should be noted that these genes are not related to the same disease. HAGH and GSS are associated with glyoxalase II deficiency (MIM 614033) and haemolytic anaemia is due to glutathione synthetase deficiency (MIM 231900). However, both inherited metabolic disorders are characterized by glyoxalase deficiency, which is a very specific clinical feature, meaning that mutations in HAGH and GSS cause low levels of glyoxalase activity. For this reason, we used the *kpath Browser* to explore all the metabolic relationships between the two enzyme-coding genes. We found that GSS and HAGH (also known as glyoxalase II or GLO2) are included in distinct Kegg pathways, glutathione (hsa00480) and pyruvate (hsa00620) metabolism, respectively. The metabolic relationships retrieved from the *kpath Browser* allow us to easily identify that both enzymes are related to the biosynthesis of glutathione. Indeed, previous studies have concluded that GSS deficiency implies low glutathione levels, which is an essential cofactor for HAGH and this could explain a deficient activity of glyoxalase (39). This suggests that both enzymes have a relevant role in glutathione metabolism (KEGG, hsa00480). However, HAGH is not considered in this pathway despite its potential contribution and dependence on glutathione levels. In addition, other authors have even considered glyoxalase as an independent pathway that has been conserved in different species. This example illustrates how to study a molecular mechanism involving two genes that are phenotypically related (Figure 4A).

In a second example, we also studied a multifactorial and prevalent phenotype such as hypercholesterolemia. We used a similar approach as in the previous case, which consists in studying all the biochemical relationships between genes that are not associated with the same genetic disease but their mutations cause similar phenotypes. We found that mutations in lipase A (LIPA) and lipoprotein lipase (LPL) cause symptoms such as nodular changes affecting the eyelids, hypercholesterolemia, hepatosplenomegaly and xanthomatosis. In addition, LIPA and LPL can be also found in different KEGG pathways (Figure 4B).

## Conclusion

This article has described a database which integrates pathway data from four different databases. We have also presented the *kpath Browser*, a client interface, which covers end-users’ needs such as navigating easily through the integrated data stored in a SPARQL Endpoint. The publication of these data as an Open SPARQL endpoint allows third-parties to develop different user interfaces on top of this database. Additionally, the integration process is based on standard Linked Data technologies enabling the integration of additional data sources with a controlled cost to improve the data offered. This process is also updatable, that is, the integration process will detect changes in the sources of information and will update the SPARQL Endpoint. Thus, users will always be accessing up-to-date integrated data

The *kpath* Browser is provided with a user interface that integrates three useful tools: the Pathway Graphical Viewer, the Pathway Graphical Editor and the Relationship Search Tool. Their combination provides users with not only a browsing interface, but also some analytical features to discover new knowledge from the integration of public data. Furthermore, the use cases described show that this tool can be useful for extracting relevant biological information. The use cases show the benefits of querying different biological concepts within a common information space. This issue becomes even more complex if one considers the multitude of available datasets and their integration. For this reason, new tools for exploring the whole set of biochemical reactions between metabolic components, like those provided by the *kpath Browser*, represent a useful step towards more accessible platforms for accessing integrated data.

## Supplementary data

Supplementary data are available at *Database* Online.

## Funding

Supported by Grants TIN2014-58304-R and TIN2011-25840 (Ministerio de Ciencia e Innovación) and P11-TIC-7529 and P12-TIC-1519 (Plan Andaluz de Investigación, Desarrollo e Innovación), and funds from Group BIO-267 (Andalusian Government and FEDER). The “CIBER de Enfermedades Raras” is an initiative from the ISCIII (Spain).

*Conflict of interest*. None declared.

## Supplementary Material

Supplementary Data
