# Supplementary material for: kpath: integration of metabolic pathway linked data
Source: Database (Oxford). 2015 Jun 8;2015:bav053. doi: 10.1093/database/bav053 (PMC4460419; doi:10.1093/database/bav053)
Supplement: Supplementary Data [file supp_2015_bav053_index.html]

kpath: integration of metabolic pathway linked data — Supplementary Data 

# kpath: integration of metabolic pathway linked data

## Supplementary Data

files

- Supplementary Data - docx file
