# Supplementary material for: kpath: integration of metabolic pathway linked data
Source: Database (Oxford). 2015 Jun 8;2015:bav053. doi: 10.1093/database/bav053 (PMC4460419; doi:10.1093/database/bav053)
Supplement: Supplementary Data [file supp_bav053_S1.docx]

**Suplementary Material S1**

- Reference: Information provided by sources (or multiple sources) that constructs a metabolic network.
- Pathway Editor Tools: Tools to edit metabolic networks (e.g., building networks from scratch, graph personalization).
- Multiple species: functionality of networks constructed from broad information about organisms.
- Facilitate sharing across group members: Allows other users to improve the application or the information included in it.
- Attached source information on nodes and edges: Source resources of pathway information to be accessed by users.
- Manipulate visual properties of nodes and edges: capability to manipulate edges and nodes at the visual properties level (e.g., different shapes to represent biochemical reactions, enzymes, metabolites etc.).
- Pathway alignment/comparison: The application enables to compare information from metabolic pathways (e.g., compare two different reactions and determine the shared nodes).
- Multiple linked views: The application displays multiple linked views for multiple conditions (e.g., pathways that link to other pathways).
- Zooming: The app allows users to zoom in on the visualization area.
- Query Pathways: The app allows specific queries to retrieve pathway information.
- Genetic information on pathways: Genetic information on genes related with pathways.
- Build history of pathway elements: This feature allows the user to track the pathways that they have edited.
- Update Database: This feature that enables the pathway database to be updated.
- Building networks from node lists by search: Construction of metabolic networks from a node list(s) using a search method. This is applicable to pathways, genes etc.
- Integration of updated data from multiple sources: This feature allows users to construct metabolic networks whose information comes from multiple sources.
- Export to standard formats: This feature allows users to export pathways in standard format files.
